# Auto-induction of phase I and phase II metabolism of artemisinin in healthy Chinese subjects after oral administration of a new artemisinin-piperaquine fixed combination

**DOI:** 10.1186/1475-2875-13-214

**Published:** 2014-06-03

**Authors:** Meitong Zang, Fanping Zhu, Xinxiu Li, Aijuan Yang, Jie Xing

**Affiliations:** 1 School of Pharmaceutical Sciences, Shandong University, 44# West Wenhua Road, Jinan 250012, P R China

**Keywords:** Artemisinin, Auto-induction metabolism, Healthy Chinese, CYP activity

## Abstract

**Background:**

Artequick is a relatively inexpensive artemisinin (Qing-hao-su; QHS)-based combination therapy (ACT) that contains QHS and piperaquine (PQ), which has not been widely used because of the decreased concentration level of QHS after repeated oral administrations for five to seven days as a monotherapy. This study was designed to evaluate the potential auto-induction metabolism of QHS in healthy Chinese adults after a two-day oral administration of QHS-PQ. The effect of QHS-PQ on the activity of the CYP2B6 and CYP3A4 was also investigated.

**Methods:**

Fourteen healthy Chinese subjects received two-day oral doses of QHS-PQ (Artequick). A two-drug cocktail consisting of bupropion and midazolam was used to assess the activities of CYP2B6 and CYP3A, respectively. Plasma samples were analysed for QHS and its phase I/II metabolites, probe drugs and their metabolites, using a validated liquid chromatography tandem mass spectrometric (LC-MS) method.

**Results:**

Four major phase I metabolites of QHS (M1-M3 and deoxy-QHS) and two subsequent phase II metabolites (M4-M5) were detected in human plasma after oral administrations of QHS-PQ. The AUC_*0-t*_ of the QHS and its phase I metabolites decreased significantly (*P* < 0.05) with increased oral clearance (CL/F) after two-day oral doses of QHS-PQ, whereas its phase II metabolites exhibited higher AUC (*P* < 0.01). The phase I metabolic capability, calculated by the AUC_*0-t*_ ratio of all phase I metabolites to QHS, increased 1.5-fold after the repeated dose (*P* < 0.01), and the phase II metabolic capability increased 1.5-fold for M4 and 3.0-fold for M5. The enzyme activity of CYP2B6 and CYP3A4 increased 2.1-fold and 3.2-fold, respectively, after two-day oral doses of QHS-PQ.

**Conclusions:**

The auto-induction of both phase I and phase II metabolism of QHS was present in healthy Chinese subjects after a recommended two-day oral dose of QHS-PQ. The auto-induction metabolism also existed for phase I metabolites of QHS. The enzyme activity of CYP2B6 and CYP3A4 was induced after the two-day oral doses of QHS-PQ. Based on these results, the alternative common three-day regimen for QHS-PQ could probably lead to lower bioavailability of QHS and higher potential of drug-drug interaction caused by the induction of drug-metabolizing enzymes.

## Background

Artemisinin (QHS) and its semisynthetic derivatives are the most potent anti-malarials available for treatment of falciparum malaria infections. Artemisinin-based combination therapy (ACT) is the recommended treatment for uncomplicated *Plasmodium falciparum* malaria by World Health Organization (WHO). The present forms of ACT contain artemether (ARM) plus lumefantrine (Coartem^®^), artesunate (ARS) plus either amodiaquine, mefloquine or sulphadoxine-pyrimethamine, and dihydroartemisinin (DHA) plus piperaquine (Artekin^®^). Although new sources of QHS are emerging from chemical synthesis [1] and engineered microbes [2], QHS is currently only commercially available from the plant *Artemisia annua* (Asteraceae). The production of semisynthetic derivatives of QHS adds to the manufacturing cost. Artequick^®^ (Artepharm Co Ltd, Guangzhou, China) is a recently marketed and relatively inexpensive ACT, which contains QHS instead of its derivatives plus piperaquine (PQ). The current manufacturer's recommendation for Artequick is a two-day regimen, which contrasts with the three-day recommended for all ACT by WHO. The simplified dosing scheme (two-day therapy) of this QHS-PQ combination may increase patient compliance and consequently the efficacy of the treatment. However, a shorter treatment regimen might not be effective in some areas and may cause QHS resistance to emerge more quickly. Several previous reports suggested that a three-day course of QHS-PQ deserves further evaluation as an alternative treatment for multidrug-resistant *P. falciparum* malaria [3,4].

The metabolism of QHS could be of great importance in determining the clinical efficacy and optimization of dose regimens of QHS-PQ. QHS underwent extensive metabolism, and the fraction of QHS excreted unchanged in human urine has been found less than 1% of an oral administration [5]. In rat liver microsomal (RLMs) incubates, four unknown metabolites (MW 282) of QHS were detected [6]. In another study, 11 phase I metabolites of QHS were found in human liver microsomal (HLMs) incubates, RLMs and rat plasma [7]. These hydroxylated and/or deoxyl products will undergo subsequent glucuronidation processes, and 12 phase II metabolites of QHS were detected in rat bile, urine and plasma. Four metabolites have been isolated from human urine, which include deoxy-QHS, deoxydihydroartemisinin, 9,10-dihydroxyartemisinin and so-called crystal 7 [5]. Based on the metabolic profiles of QHS *in vitro* and in rats, related phase I and phase II metabolites could exist for QHS in human, which has limited data available, especially in human blood circulation system.

QHS has not been used to a great extent in ACT because of its low bioavailability and time-dependent pharmacokinetics, which have been confirmed in healthy volunteers and infected patients as a several-fold decrease in plasma concentration of QHS with a corresponding increase in oral clearance after repeated oral administrations for five to seven days [3,8-10]. The auto-induction metabolism has been suggested for this time-dependency [11-13]. The elimination of QHS is mediated primarily by CYP2B6, with a probable secondary contribution of CYP3A4 and CYP2A6 [14,15]. QHS appears to induce several enzymes, including CYP2B6 (mepheytoin *N*-demethylation) *in vivo* and bupropion hydroxylation *in vitro*[12,13]; CYP3A4 (midazolam hydroxylation [13,16]); CYP2C19 (omeprazole as a marker; [17,18]); CYP2A6 (coumarin hydroxylation [19]); and glucuronidation (7-hydroxycoumarin glucuronidation [19]). However, the auto-induction, metabolism-mediated, time-dependent pharmacokinetics should be less pronounced if QHS was used in a combination treatment that had therapy duration shorter than that used in monotherapy. Although the tolerability, safety, efficacy, and pharmacokinetic properties of QHS have been investigated, no direct evidence was available for the increased concentration level of QHS phase I/II metabolites in blood circulation system after repeated administrations of QHS in human. The effect of QHS-PQ on the activity of the principal CYP450 enzymes (especially CYP2B6) involved in drug metabolism has not yet been studied *in vivo*.

The main objective of the current study was to evaluate the potential auto-induction metabolism of QHS during a recommended two-day oral administration of a new ACT (Artequick). The biotransformation of QHS in Chinese subjects was studied, and the pharmacokinetic profiles of QHS and its phase I/phase II metabolites were investigated. The effect of this ACT on the activity of CYP2B6 and CYP3A4 was also studied after repeated administrations.

## Methods

### Chemicals and reagents

**Figure 1:**
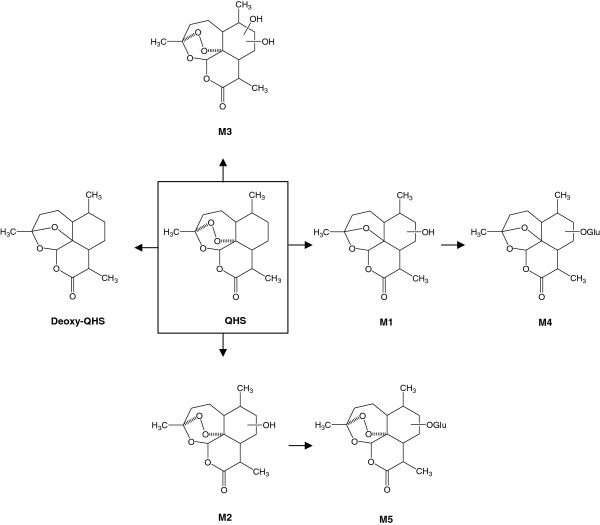
Structures of phase I (Deoxy-QHS, M1-M3) and phase II (M4, M5) metabolites of artemisinin (QHS) detected in human plasma, and proposed metabolic pathways for QHS.

Artequick tablets were provided by Artepharm Co Ltd (Guangzhou, China). QHS and ARM (as internal standard) were purchased from Kunming Pharmaceutical Co (purity >99.0%, Yunnan, China). Deoxyartemisinin (Deoxy-QHS; Figure 1) was synthesized in laboratory, and its structure was confirmed by HR-MS, ^1^H-NMR and ^13^C-NMR spectroscopy. Bupropion (BUP), (±)-hydroxybupropion (OH-BUP), midazolam (MDZ), α-hydroxymidazolam (OH-MDZ) and alprazolam were purchased from Cerilliant Corporation (Round Rock, TX, USA). All other chemicals used were purchased from Sigma-Aldrich or Thermo Fisher Scientific.

### Instrumentation

All LC-MS experiments were carried out on a Thermo Electron LTQ-Orbitrap XL hybrid mass spectrometer (ThermoFinnigan, Bremen, Germany) equipped with an electrospray ionization interface. An Accela HPLC system (ThermoElectron) was equipped with an autosampler, a vacuum degasser unit and a quaternary pump. The mass spectrometer employing positive ionization was calibrated using the manufacturer’s calibration standards mixture allowing for mass accuracies < 5 ppm in external calibration mode. The ionization voltage was 4.2 kV and the capillary temperature was set at 300°C. Nitrogen was used as both the sheath gas (40 units) and auxiliary gas (10 units). The resolving power was 15,000 for full-scan and 7,500 for the MS^2^ scans.

For metabolite identification of QHS, the data-dependent MS^2^ acquisition was triggered when the Orbitrap detected the ions of QHS metabolites on the parent ion list or the most intense ion. The [M + NH_4_]^+^ adduct ions of QHS and its metabolites were dissociated in the high collision-induced dissociation (HCD) mode using the normalized collision energy at 35% with an isolation width of 3 Da and an activation time of 30 ms. The full scan mode across an *m/z* range that bracketed QHS and its potential phase I/II metabolites was used for quantification of QHS and its metabolites. Extracted ion chromatograms (EICs) of *m/z* 283.1540 for QHS and monohydroxylated deoxy-QHS, *m/z* 284.1856 for deoxy-QHS, *m/z* 316.1775 for monohydroxylated QHS, *m/z* 332.1704 for dihydroxylated QHS, *m/z* 476.2126 for the glucuronide of monohydroxylated deoxy-QHS, *m/z* 492.2076 for the glucuronide of monohydroxylated QHS, and *m/z* 316.2118 for ARM (the internal standard; IS), with a 5 ppm range centred on the exact *m/z* value were generated for quantitation. Chromatographic separation was achieved on a Luna ODS C18 column (150 × 2.1 mm i d, 5 μm; Phenomenex, Torrance, CA, USA) with a 4.0 × 3.0 mm i d SecurityGuard C18 (5 μm) guard column (Phenomenex, Torrance, CA, USA). The chromatography was performed at 22°C. The mobile phase consisted of acetonitrile and 5 mM aqueous ammonium acetate containing 0.1% (v/v) formic acid, delivered at a flow rate of 0.35 mL/min. The gradient chromatographic conditions were shown in the previous report [7]. The autosampler was set at 4°C.

For quantification of probe drugs (BUP, MDZ) and their respective metabolites (OH-BUP, OH-MDZ), the high-resolution full scan mode was used. Extracted ion chromatograms (EICs) of *m/z* 326.0855 for MDZ, *m/z* 342.0804 for OH-MDZ, *m/z* 240.1150 for BUP, *m/z* 256.1099 for OH-BUP, and *m/z* 309.0902 for alprazolam (IS), with a 5 ppm range centred on the exact *m/z* value were generated for quantitation. Chromatographic separation was achieved on an Agilent Eclipse XDB C18 column (150 × 4.6 mm i d, 5 μm; Agilent Technologies, Santa Clara, CA, USA) with a 4.0 × 3.0 mm i d SecurityGuard C18 (5 μm) guard column (Phenomenex, Torrance, CA, USA). The chromatography was performed at 22°C. The mobile phase consisted of B (acetonitrile) and A (0.1% formic acid in water), which increased linearly from 20% B to 90% B during 7 min before column re-equilibration at a flow rate of 0.3 mL/min.

### Metabolite identification of QHS

1 mL of pooled human plasma (n = 6) or urine samples were loaded onto pretreated solid-phase extraction cartridges (Oasis extraction cartridges, Waters Corp, Milford, MS, USA). The cartridges were washed with 1 mL of water and then eluted with 0.5 mL of methanol. The methanol fractions were dried and reconstituted with 200 μL of initial mobile phase. Aliquot (20 μL) of the reconstituted solutions were injected onto LC-HR/MS.

### Quantification of QHS and its phase I/II metabolites

Plasma samples were subjected to a protein precipitation extraction process, which was performed on ice. A 100 μL aliquot of human plasma was mixed with 25 μL of acetonitrile and 200 μL of IS (1 μM of ARM, prepared in acetonitrile). The mixture was mixed and centrifuged at 3,000 g for 10 min. Aliquots (20 μL) of the solution were injected onto LC-MS analysis. For calibration preparation, 100 μL of drug-free plasma was mixed with 25 μL of stock solution (QHS and deoxy-QHS) and 200 μL of IS. This mixture was treated as above. The calibration graph was plotted by least-squares linear regression of the peak-area ratios (QHS or deoxy-QHS to IS) against concentrations of QHS or deoxy-QHS. Matrix matched calibration standards were obtained with concentrations of 17.7-709.2 nM for QHS and 18.8-751.9 nM for deoxy-QHS in plasma. QC samples were obtained with three concentration levels (35.5, 177.3 and 567.4 nM for QHS; 37.6, 188.0 and 601.5 nM for deoxy-QHS) in plasma.

Due to unavailability of reference substances, quantitative data of the other three phase I metabolites (M1-M3) and two phase II metabolites (M4-M5) were extracted based on the calibration curve of QHS. In this study, the pharmacokinetic profile of each metabolite in humans after a single oral dose of QHS-PQ will be used to compare with that after a repeated oral dose, in order to evaluate the auto-induction metabolism. In this case, the response factor for QHS metabolites compared with that of QHS was not considered. Plasma samples were diluted with blank plasma and reanalysed when the concentration of QHS metabolites was higher than the upper limit of quantification of QHS.

### Quantification of probe drugs and their metabolites

Plasma samples were subjected to a protein precipitation extraction process. A 100 μL aliquot of human plasma was mixed with 25 μL of acetonitrile and 200 μL of IS (1 μM of alprazolam, prepared in acetonitrile). The mixture was mixed and centrifuged at 3,000 g for 10 min. Aliquots (20 μL) of the solution were injected onto LC-MS analysis. For calibration preparation, 100 μL of drug-free plasma was mixed with 25 μL of stock solution (BUP, OH-BUP, MDZ and OH-MDZ) and 200 μL of IS. This mixture was treated as above. The calibration graph was plotted by least-squares linear regression of the peak-area ratios (BUP, OH-BUP, MDZ or OH-MDZ to IS) against concentrations of BUP, OH-BUP, MDZ or OH-MDZ. Matrix matched calibration standards were obtained with concentrations of 7.7-307.7 nM for MDZ, 1.5-58.7 nM for OH-MDZ, 4.2-836.8 nM for BUP, and 19.6-3,921.6 nM for OH-BUP in human plasma. QC samples were obtained with three concentration levels (15.4, 76.9 and 246.2 nM for MDZ; 2.9, 14.7 and 46.9 nM for OH-MDZ; 8.4, 83.7 and 669.5 nM for BUP; 39.2, 392.2, 3,137.3 nM for OH-BUP) in plasma. Plasma samples were diluted with blank plasma and reanalysed when the concentration of any analyte was higher than the upper limit of quantification.

### Method validation

Two LC-MS methods were developed for quantification of QHS metabolites and probe drugs. The method was evaluated through linearity, intra- and inter-day precision and accuracy. The accuracy and precision of the method were assessed by determining QC samples using six replicated preparations of plasma samples at three QC concentration levels on three separate days. The lower limit of quantification (LLOQ) represents the lowest concentration of the analyte over the linear range that can be determined with acceptable precision and accuracy.

Bench-top stability of QHS and deoxy-QHS was assessed by leaving the QC samples of two different concentrations (35.5 and 567.4 nM for QHS; 37.6 and 601.5 nM for deoxy-QHS) on ice for 2 hours. The stability of QHS samples in autosampler vials was assessed at 4°C for 12 hours. The stability of probes drugs (BUP, MDZ) and their metabolites (OH-BUP, OH-MDZ) were also evaluated.

### Drug administration and sample collection

The experiment followed guidelines of the Declaration of Helsinki for humans, and the experimental protocol was approved by the Ethics Committee of Shandong University (Jinan, China) and the Institutional Review Board of Qilu Hospital (Shandong University, China). The clinical project was performed at Qilu Hospital (Jinan, China). Fourteen healthy and non-smoking male volunteers (18–24 years; body mass index of 19–24 kg/m^2^) were enrolled in the clinical trial, and written informed consent was provided prior to enrolment in this study. They were in good health as assessed by medical history, physical examination and laboratory analysis (complete blood count, total bilirubin, direct bilirubin, serum creatinine, blood urea nitrogen, alanine aminotransferase, and serum albumin). The subjects were fasted overnight before drug administration and for a further 2 hours after dosing. Water was freely available during experiments.

Each subject was treated with QHS-PQ tablets (Artequick) according to manufacturer’s recommendation (125 mg of QHS plus 750 mg of PQ each day for two consecutive days). Venous blood samples (2 mL) for determination of QHS and its metabolites were taken from an indwelling intravenous catheter at 0, 0.25, 0.75, 1.25, 1.75, 2.5, 3.0, 4.0, 5.0, 6.0, 8.0, 12.0, 24.0, 36.0 (second dose), and 48.0 hours (second dose) after each dose, and collected in anticoagulant tubes drawn from forearm venous catheters before and after oral administration of QHS-PQ. Plasma was separated by centrifugation at 3,000 g for 10 min at 4°C. The plasma was stored at -80°C until analysis.

The probe drugs, including midazolam (15 mg tablet, Nhwa Pharma Corp, China) and bupropion (75 mg tablet, Venturepharm Pharmaceutical Co Ltd, Hainan, China) were given orally two weeks before (day -14) administration of QHS-PQ. The probe drug cocktail was repeated 1 hour after intake of the second oral dose of QHS-PQ (day 2). Venous blood samples (2 mL) for analysis of probe drugs and their metabolites were taken from an indwelling intravenous catheter at 0, 0.25, 0.75, 1.5, 2.0, 3.0, 4.0, 5.0, 7.0, 11.0, 24.0, 36.0, and 48.0 hours.

### Data processing

The peak plasma concentration (*C*_max_) and time-to-peak concentration (*T*_max_) were obtained from experimental observations. The other pharmacokinetic parameters were analysed by non-compartmental model using the program TOPFIT (version 2.0; Thomae GmbH, Germany). The area under the plasma concentration-time curve (AUC_*o-t*_) was calculated using the linear trapezoidal rule to approximately the last point. The mean residence time (MRT) was obtained by dividing the area under the first moment-time curve (AUMC_*0-t*_) by the area under the curve (AUC_*0-t*_). Total oral body clearance (CL/F) was calculated as dose/AUC_*0-t*_. The inter-individual variability of pharmacokinetic parameters of QHS was calculated by the difference between individual values and mean values. The metabolic capability leading to phase I metabolites of QHS was calculated by the AUC_*0-t*_ ratio (AUC_*phase I metabolite*_/AUC_*QHS*_) of phase I metabolites to the parent drug QHS, and the metabolic capability to form phase II metabolites of QHS was evaluated by the AUC_*0-t*_ ratio (AUC_*phase II metabolite*_/AUC_*phase I metabolite*_) of phase II metabolites to their respective hydroxylated metabolites. Induction of phase I metabolism by QHS was assessed by comparison of the AUC_o-∝_ ratio of all phase I metabolites to the parent drug QHS between one-day and two-day oral doses of QHS-PQ. Induction of phase II metabolism by QHS was evaluated by comparison of the AUC_*o-t*_ ratio of phase II metabolites to their respective hydroxylated metabolites between two doses. The enzyme activity of CYP2B6 was calculated by the AUC_*o-∝*_ ratio of OH-BUP to BUP, and the CYP3A4 activity was determined by the AUC_*o-∝*_ ratio of OH-MDZ to MDZ.

Results were expressed as mean ± SD. The two-tailed *t*-test was used for paired comparison of the pharmacokinetic parameters between the single dose and multiple doses after logarithmic transformation. Geometric mean ratios with 90% confidence intervals (CIs) were calculated after log transformation of within-subject data. The comparison of *T*_max_ for the different treatment groups was performed using the Wilcoxon signed-rank test. The acceptable level of significance was established at *P <* 0.05 or *P* < 0.01. A greater than 1.2-fold increase in AUC/dose or CYP activity, relative to the control, was defined to be induction.

## Results

### Metabolite identification of QHS

**Figure 2:**
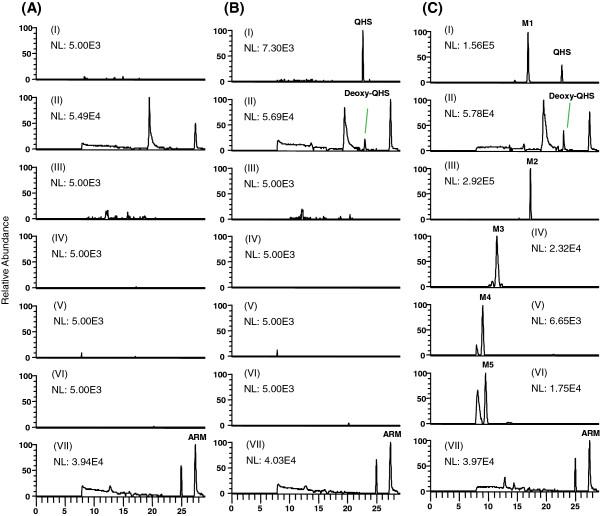
**Representative full-scan chromatograms of (A) a blank human plasma sample spiked with IS (ARM, 1 μM); (B) a blank human plasma sample spiked with artemisinin (QHS; 17.7 nM), deoxyartemisinin (Deoxy-QHS; 18.8 nM), and IS (ARM, 1 μM); and (C) a human plasma sample at 1.25 h after a single oral dose of QHS (1.8 mg/kg).** I: QHS and monohydroxylated deoxyartemisinin (*m/z* 283.1540); II: deoxyartemisinin (deoxy-QHS; *m/z* 284.1856); III: monohydroxylated artemisinin (*m/z* 316.1775); IV: dihydroxylated artemisinin (*m/z* 332.1704) V: the glucuronide of monohydroxylated deoxy-QHS M5 (*m/z* 476.2126); VI: the glucuronide of monohydroxylated QHS (*m/z* 492.2076); VII: IS (ARM, *m/z* 316.2118).

Human plasma and urine samples were analysed by liquid chromatography-high resolution mass spectrometry (LC-HRMS), as illustrated in selected ion chromatograms of QHS, its four major phase I metabolites and two major phase II metabolites in human plasma as a representative (Figure 2). Based on the accurate MS and MS/MS spectra, six major metabolites in human plasma were proposed to be monohydroxylated deoxy-QHS (M1), deoxy-QHS, monohydroxylated QHS (M2), dihydroxylated QHS (M3), the glucuronide of monohydroxylated deoxy-QHS (M4) and the glucuronide of monohydroxylated QHS (M5), respectively. Their proposed structures are shown in Figure 1. Five more phase I metabolites were detected in human urine, including three monohydroxylated deoxy-QHS and two monohydroxylated QHS (not shown). The identification of phase I and phase II metabolites of QHS *in vitro* and in rats using LTQ/Orbitrap has been shown in detail in a previous study [7]. In the present study, the pharmacokinetics of QHS and its six major metabolites (M1-M5, and deoxy-QHS) in human plasma was studied.

### LC-MS method for determination of QHS and its metabolites

Under optimized HPLC conditions, QHS and its phase I/II metabolites were eluted within 30 min (Figure 2). Blank human plasma from six lots showed no significant interfering peaks at the retention times of each analyte (Figure 2A). The LC separation was improved allowing the separation of QHS metabolites from their minor diastereomers.

The calibration curves of QHS and deoxy-QHS were linear over the concentration range of 17.7-709.2 nM and 18.8-751.9 nM, respectively, in human plasma with correlation coefficients *r* > 0.99 and consistent slope values when evaluated by weighted (1/*x*^2^) least-squares linear regression. The LLOQ of QHS and deoxy-QHS in human plasma were established at 17.7 and 18.8 nM, respectively. The precision and accuracy of this method indicate that all coefficients of variation at each concentration level were below 15%. There was no significant difference (< 15%) between the responses of standards at time zero and after storage of plasma on ice for at least 2 hours in terms of %CV for QHS and deoxy-QHS, indicating that QHS and deoxy-QHS were stable under this condition. Processed samples were stable for at least 12 hours in the autosampler tray (4°C).

### Pharmacokinetics of QHS and its phase I metabolites

**Figure 3:**
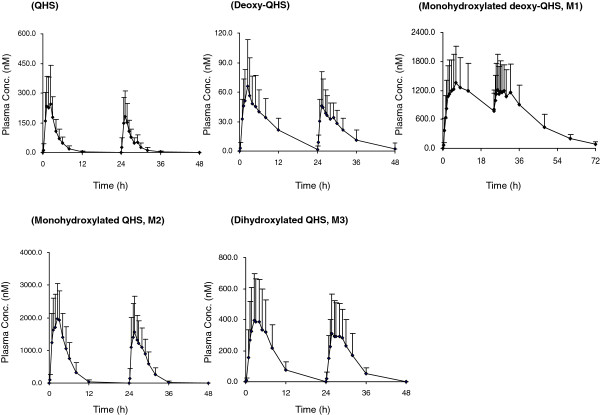
Mean (+SD) plasma concentration-time profiles of artemisinin (QHS) and its phase I metabolites (deoxyartemisinin: Deoxy-QHS; monohydroxylated deoxy-QHS: M1; monohydroxylated QHS: M2; dihydroxylated QHS: M3) in healthy Chinese (n = 14 each) following two consecutive days of oral doses of QHS (1.8 mg/kg).

The mean plasma concentration-time profiles of QHS and its phase I metabolites (M1-M3, deoxy-QHS) after oral dosing of QHS are shown in Figure 3, and the pharmacokinetic parameters are given in Table 1.

**Table 1:** The main pharmacokinetic parameters of artemisinin (QHS) and its phase I/II metabolites (deoxy-QHS, monohydroxylated deoxy-QHS, monohydroxylated QHS, dihydroxylated QHS, the glucuronide of monohydroxylated deoxy-QHS, and the glucuronide of monohydroxylated QHS) after one-day and two-day oral administrations of QHS-PQ (1.8 mg/kg of QHS plus 10.6 mg/kg piperaquine) to 14 healthy Chinese subjects (mean ± sd)

		**AUC**_**0-t**_**/dose (h·kg·L**^**-1**^**)**	**C**_**max **_**(nmol·L**^**-1**^**)**	***T***_**max **_**(h)**	**MRT (h)**	**CL/F (L/h/kg)**	**AUC**_**M**_**/AUC**_**QHS**_
1-day	QHS	0.14 ± 0.08**	304.3 ± 191.7	1.8 (0.75, 3.0)	3.2 ± 0.9	9.81 ± 8.25**	N.A.
	DeoxyQHS	0.09 ± 0.05**	71.0 ± 46.1*	2.2 (1.25, 4.0)	7.1 ± 0.6	14.71 ± 8.41**	0.67 ± 0.25
	M1	3.72 ± 1.59**	1527.4 ± 721.7	7.0 (3.0, 12.0)	11.6 ± 0.7**	0.36 ± 0.27**	28.80 ± 11.31**
	M2	1.66 ± 0.83**	2378.5 ± 1135.6**	2.1 (1.25, 3.0)	4.0 ± 0.6	0.80 ± 0.52**	12.61 ± 4.56
	M3	0.49 ± 0.30*	451.6 ± 299.2	3.3 (1.25, 6.0)	5.7 ± 1.2	7.55 ± 15.62*	3.80 ± 2.90
	M4	0.02 ± 0.01**	22.8 ± 17.1**	1.9 (0.75, 3.0)	4.4 ± 2.0	135.13 ± 141.70**	0.007 ± 0.01**†
	M5	0.02 ± 0.01**	58.7 ± 37.7**	1.7 (0.75, 3.0)	2.4 ± 0.8	82.54 ± 66.47**	0.02 ± 0.02**‡
2-day	QHS	0.09 ± 0.05**	221.4 ± 117.9	1.4 (0.75, 1.75)	2.9 ± 0.9	16.60 ± 14.79**	N.A.
	DeoxyQHS	0.06 ± 0.04**	55.6 ± 33.3*	2.3 (1.25. 5.0)	6.2 ± 1.5	24.71 ± 20.75**	0.79 ± 0.47
	M1	3.15 ± 1.42**	1382.3 ± 680.6	3.5 (1.25, 8.0)	9.8 ± 0.7**	0.43 ± 0.30**	43.08 ± 27.12**
	M2	1.25 ± 0.62**	1973.9 ± 1084.4**	2.0 (1.25, 5.0)	3.9 ± 0.6	1.15 ± 0.92**	16.91 ± 10.18
	M3	0.39 ± 0.26*	366.3 ± 250.0	3.0 (1.25, 5.0)	5.6 ± 1.3	4.43 ± 5.00*	5.82 ± 6.28
	M4	0.03 ± 0.02**	35.4 ± 15.7**	1.9 (0.75, 4.0)	4.7 ± 1.5	51.54 ± 31.52**	0.01 ± 0.01**†
	M5	0.04 ± 0.01**	99.9 ± 51.7**	1.5 (0.75, 4.0)	2.7 ± 0.5	29.85 ± 11.98**	0.05 ± 0.05**‡
Geometric mean ratios (90% CI)	QHS	0.64 (0.56, 0.73)**	0.73 (0.56, 0.94)	N.A.	0.92 (0.78, 1.09)	1.69 (1.48, 1.94)**	N.A.
	DeoxyQHS	0.68 (0.58, 0.79)**	0.78 (0.69, 0.89)*	N.A.	0.89 (0.79, 0.99)	1.68 (1.45, 1.95)**	1.18 (0.97, 1.43)
	M1	0.85 (0.79, 0.91)**	0.90 (0.80, 1.03)	N.A.	0.84 (0.81, 0.88)**	1.18 (1.10, 1.26)**	1.50 (1.31, 1.71)**
	M2	0.75 (0.68, 0.83)**	0.83 (0.73, 0.95)**	N.A.	0.98 (0.89, 1.07)	1.43 (1.30, 1.58)**	1.34 (1.18, 1.53)
	M3	0.79 (0.44, 1.40)*	0.82 (0.70, 0.96)	N.A.	0.96 (0.89, 1.04)	0.59 (0.33, 1.05)*	1.53 (1.13, 2.07)
	M4	1.66 (1.30, 2.12)**	1.55 (1.26, 1.91)**	N.A.	1.07 (0.91, 1.26)	0.38 (0.30, 0.49)**	1.45 (1.13, 1.88)**†
	M5	1.98 (1.62, 2.41)**	1.70 (1.41, 2.05)**	N.A.	1.12 (0.98, 1.29)	0.36 (0.30, 0.44)**	2.95 (2.43, 3.58)**‡

**: *P* < 0.01; *: *P* < 0.05 (single dose compared with multiple dose); †: AUC_M4_/AUC_M1_; ‡:AUC_M5_/AUC_M2_; *N.A*., not acquired.

Healthy subjects in this study received a recommended lower dose of QHS (1.8 mg/kg/day). After the first dose, QHS was rapidly eliminated, with a high mean CL/F of 9.8 L/h/kg and a short mean MRT of 3.2 hours. The dose-normalized AUC value of QHS was 0.14 ± 0.08 h·kg/L after a single oral dose of QHS. There was also a large inter-individual variability in QHS pharmacokinetic parameters, with a %CV of 43.5% (6.9-122.7%) for AUC_*0-t*_. The time-dependent pharmacokinetics existed for QHS, and the second oral dose of QHS-PQ resulted in a 35.2% decrease in AUC_*0-t*_ compared with the first dose. The corresponding CL/F value significantly increased 1.7-fold (90% CI, 1.5-1.9). The *C*_max_, *T*_max_ and MRT of QHS did not change (*P* > 0.05) after repeated drug dosing.

**Figure 4:**
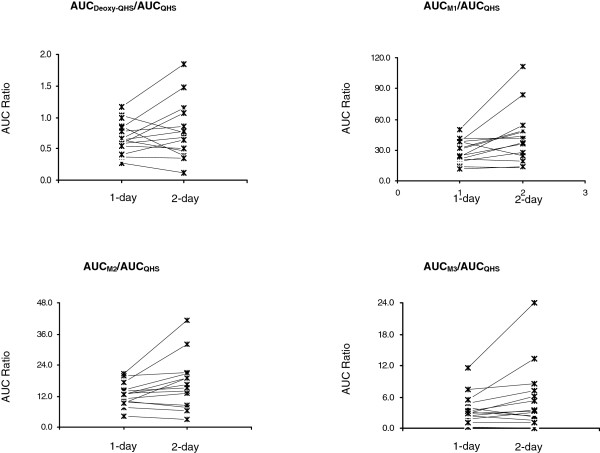
Individual values for AUC ratio of phase I metabolites (M1-M3 and deoxy-QHS) of artemisinin (QHS) to the parent drug in healthy Chinese (n = 14 each) following one-day or two-day oral doses of QHS (1.8 mg/kg).

The AUC_*0-t*_ of the metabolite deoxy-QHS decreased significantly (*P* < 0.01) by 34.7% (20.3-71.8%) in 13 out of 14 subjects with increased CL/F values (1.7-fold), after multiple oral doses of QHS. The other pharmacokinetic parameters (*T*_max_ and MRT) did not change (*P* > 0.05). The metabolic capability, calculated by AUC_deoxy-QHS_/AUC_QHS_, increased 1.6-fold in six subjects after the second dose but decreased by 41.6% in four subjects (Figure 4).

The metabolite M1, monohydroxylated deoxy-QHS, was present in relatively high level (mean *C*_max_ of 1.5 μM). Its *C*_max_ was reached (7.0 (3.0-12.0 hours)) later than that of QHS (0.8-3.0 hours) and deoxy-QHS (1.3-4.0 hours). The second dose of QHS-PQ only resulted in a minor decrease (14.5%) in AUC_*0-t*_ of M1 (*P* < 0.01). The metabolic capability, calculated by AUC_M1_/AUC_QHS_, increased 1.8-fold (1.4-2.3) in eight subjects and kept unchanged in four subjects (Figure 4).

Monohydroxylated QHS (M2) was observed in relatively high level (mean *C*_max_ of 2.4 μM). The repeated dose of QHS led to a significant (*P* < 0.01) decrease by 25.0-51.5% in AUC_*0-t*_ of M2 in nine subjects. The ratio of AUC_M2_/AUC_QHS_ increased 1.6-fold (1.2-2.0) in seven subjects and kept unchanged in five subjects (Figure 4).

The metabolite dihydroxylated QHS (M3) was proposed to be 9,10-dihydroxyQHS based on a previous report [5]. The AUC_*0-t*_ of M3 decreased significantly (*P* < 0.01) in seven subjects after the repeated dose, whereas the ratio of AUC_M3_/AUC_QHS_ increased 1.9-fold (1.5-2.5) in seven subjects and decreased in four subjects (Figure 4).

Compared with one-day oral dosing of QHS-PQ, the higher AUC ratio [1.5-fold (90% CI, 1.3-1.7)] of total phase I metabolites to QHS was observed after two-day oral doses (*P* < 0.01).

### Pharmacokinetics of phase II metabolites of QHS

**Figure 5:**
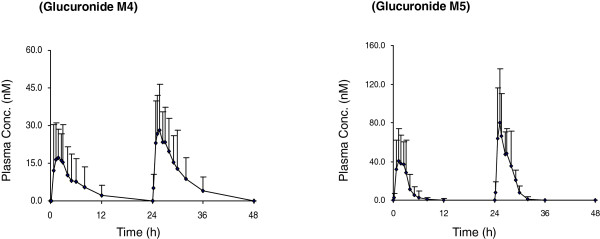
Mean (+SD) plasma concentration-time profiles of phase II metabolites of artemisinin (QHS) (the glucuronide of monohydroxylated deoxy-QHS: M4; the glucuronide of monohydroxylated QHS: M5) in healthy Chinese (n = 14 each) following two consecutive days of oral doses of QHS (1.8 mg/kg).

After QHS-PQ was administrated to healthy Chinese, two major phase II metabolites (M4 and M5) of QHS were detected (Figure 2), and they were supposed to be the glucuronidation products of monohydroxylated deoxy-QHS (M1) and monohydroxylated QHS (M2), respectively. The AUC ratios of M4 to M1 and M5 to M2 were used to evaluate the potential induction of phase II metabolism. Their mean plasma concentration-time profiles are shown in Figure 5. The pharmacokinetic parameters are presented in Table 1.

**Figure 6:**
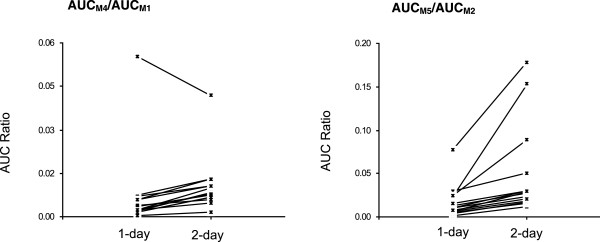
Individual values for AUC ratio of phase II metabolites (M4 and M5) to their respective hydroxylated metabolites (M1, M2) of artemisinin (QHS) in healthy Chinese (n = 14 each) following one-day or two-day oral doses of QHS (1.8 mg/kg).

Time-dependent pharmacokinetics was also observed for these phase II metabolites after a two-day oral dose of QHS-PQ. Higher AUC_*0-t*_ and *C*_max_ values were found for both M4 (except one subject) and M5 after multiple oral doses. The dose-normalized AUC_*0-t*_ increased 1.7-fold for M4, and the metabolic capability (AUC_M4_/AUC_M1_) increased 2.9-fold (1.5-4.5) in 13 subjects after the repeated dosing (Figure 6). Similarly, the dose-normalized AUC_*0-t*_ increased 2.0-fold (90% CI, 1.6-2.4) for M5, and the metabolic capability (AUC_M5_/AUC_M2_) increased 3.0-fold (90% CI, 2.4-3.6) after two days’ oral administration of QHS-PQ (Figure 6).

The corresponding CL/F of two phase II metabolites decreased significantly (*P* < 0.01) after the second dose. Their *T*_max_ and MRT did not change significantly (*P* > 0.05) after repeated drug dosing.

### The effect of QHS-PQ on the enzyme activity

After an oral administration of two probe drugs (BUP and MDZ), the dose-normalized AUCs of BUP (0.35 ± 0.23 h·kg/L), OH-BUP (3.98 ± 1.45 h·kg/L), MDZ (1.0 ± 0.3 h·kg/L) and its metabolite OH-MDZ (0.32 ± 0.09 h·kg/L) were obtained. The metabolic capability was 13.4 ± 5.2 for CYP2B6 (AUC ratio of OH-BUP to BUP) and 0.34 ± 0.09 for CYP3A4 (AUC ratio of OH-MDZ to MDZ). The enzyme activity of CYP2B6 (bupropion hydroxylation) and CYP3A (midazolam hydroxylation) increased 2.1-fold (90% CI, 1.8-2.3) and 3.2-fold (90% CI, 2.8-3.7), respectively, after two days’ oral intake of QHS-PQ compared with day -14. The induction of CYPs activity by QHS-PQ is summarized in Table 2.

**Table 2:** The effect of artemisinin (QHS) on the activity of CYP2B6 (bupropion hydroxylation) and CYP3A4 (midazolam hydroxylation) in humans

		**AUC**_**0-t**_**/dose (h·kg·L**^**-1**^**)**	**C**_**max **_**(nmol·L**^**-1**^**)**	**T**_**max **_**(h)**	**MRT (h)**	**CL/F (L/h/kg)**
BUP	1-day	0.35 ± 0.23	327.86 ± 180.54	2.1 (1.5, 4.0)	6.8 ± 1.9	3.73 ± 1.91
	2-day	0.34 ± 0.18	305.93 ± 146.69	2.5 (0.8, 5.0)	6.1 ± 1.7	3.78 ± 2.05
	Geometric mean ratios (90% CI)	0.96 (0.83, 1.12)	0.93 (0.77, 1.13)	N.A.	0.89 (0.80, 1.00)	1.01 (0.87, 1.17)
OH-BUP	1-day	3.98 ± 1.45**	517.77 ± 162.04**	5.2 (3.0, 11.0)	19.4 ± 1.4	0.29 ± 0.11**
	2-day	8.22 ± 3.43**	856.60 ± 245.03**	7.6 (3.0, 24.0)	20.2 ± 1.5	0.14 ± 0.05**
	Geometric mean ratios (90% CI)	2.05 (1.83, 2.30)**	1.65 (1.51, 1.82)**	N.A.	1.04 (1.00, 1.07)	0.49 (0.43, 0.55)**
MDZ	1-day	1.00 ± 0.33**	206.03 ± 114.68*	1.3 (0.3, 2.0)	4.1 ± 0.7	1.09 ± 0.33**
	2-day	1.55 ± 0.42**	305.26 ± 114.80*	0.8 (0.3, 3.0)	4.2 ± 0.7	0.70 ± 0.21**
	Geometric mean ratios (90% CI)	1.55 (1.35, 1.78)**	1.48 (1.12, 1.97)*	N.A.	1.04 (0.96, 1.14)	0.64 (0.55, 0.73)**
OH-MDZ	1-day	0.32 ± 0.09**	82.32 ± 50.26**	1.1 (0.3, 2.0)	3.7 ± 1.0	3.31 ± 0.84**
	2-day	1.60 ± 0.26**	242.64 ± 81.98**	1.5 (0.8, 3.0)	4.2 ± 0.5	0.64 ± 0.11**
	Geometric mean ratios (90% CI)	4.99 (4.66, 5.33)**	2.95 (2.44, 3.56)**	N.A.	1.14 (1.01, 1.28)	0.19 (0.18, 0.21)**
AUC_OH-BUP_/AUC_BUP_	1-day	13.36 ± 5.24**	N.A.	N.A.	N.A.	N.A.
	2-day	27.33 ± 10.06**	N.A.	N.A.	N.A.	N.A.
	Geometric mean ratios (90% CI)	2.05 (1.79, 2.33)**	N.A.	N.A.	N.A.	N.A.
AUC_OH-MDZ_/AUC_MDZ_	1-day	0.34 ± 0.09**	N.A.	N.A.	N.A.	N.A.
	2-day	1.10 ± 0.29**	N.A.	N.A.	N.A.	N.A.
	Geometric mean ratios (90% CI)	3.24 (2.84, 3.69)**	N.A.	N.A.	N.A.	N.A.

*MDZ*: midazolam; *BUP*: bupropion; *OH-MDZ*: hydroxymidazolam; *OH-BUP*: hydroxybupropion.

*: *P* < 0.05; **: *P* < 0.01 (1-day compared with 2-day); *N.A*., not acquired.

## Discussion

For LC-MS technique, quantitation is only possible by comparison with a standard for each compound. Absolute quantification of metabolites M1-M5 could not be achieved in this case due to unavailability of reference substances. Semi-quantitative data can be extracted, for example, by comparing the integration of the peaks, from each metabolite to the area of the parent compound. The accuracy of these data, however, relies on the assumption that the response factor for each metabolite is comparable to the response factor for the parent compound. Because of ion suppression caused by matrix effects, mobile phase composition, MS detector dynamic range and modification of the structure during metabolism response factors of MS will inevitably vary between each analyte; thus, semi-quantitative data can only be viewed as a general indication of relative abundance. In this study, even though no synthetic standard was available for metabolites M1-M5, based on past experience it is reasonable to expect that these metabolites will exhibit similar ionization efficiency to the parent molecule QHS in positive ion mode. Thus, it can be deduced from these data that these metabolites are likely major circulating metabolites of QHS in healthy Chinese subjects. The pharmacokinetic profiles of QHS and its metabolites in humans after a single oral dose will be used to compare with that after multiple oral doses, to evaluate the auto-induction metabolism. In this case, the response factor for each metabolite compared with that of QHS was not considered, and quantitative data of metabolites M1-M5 could be calculated using the calibration curve of QHS.

Several published studies have evaluated the pharmacokinetics of the parent drug QHS in healthy adults and patients including children, after oral administration of QHS either as monotherapy or ACT [3,8-10]. These findings suggest that PQ should not influence the pharmacokinetic characteristics of QHS when co-administered in the proposed fixed oral combination [8]. Quantitative prediction of P450-mediated DDIs indicated auto-induction of QHS clearance with the AUC_*i*_/AUC ratio decreasing to 59% [13]. The extent of the increase in QHS CL/F has been reported to be 5- to 7-fold after five days’ oral treatment with 500 mg of QHS [10]. The relative bioavailability after four to seven days was between 0.13 and 0.29. There was also evidence of a more rapid auto-induction of QHS metabolism (the AUC of QHS decreased to 0.27) in infected children after the second dose [3]. This suggested that the higher clearance of QHS in children at critical times of malaria could lead to more failures of ACT than in adults. In this study, the pharmacokinetic profiles of QHS metabolites in humans were also evaluated, which has not been reported.

The dose-normalized AUC value (0.14 ± 0.08 h·kg/L) obtained from a single oral dose of QHS in the present study was within the range of 0.06-0.31 h·kg/L reported for healthy subjects and patients who received 500 mg of QHS [8,9], but lower than that in infected children (0.33-0.49 h·kg/L) at the dose of 3.2 mg/kg [3]. The present results showed that the time-dependent pharmacokinetics existed for QHS and its phase I and phase II metabolites even after a two-day oral dose of QHS-PQ. Out of expectation, most of phase I metabolites (deoxy-QHS, M1-M3) showed reduced concentration level, which was probably caused by the further induction metabolism and/or more rapid subsequent clearance of those metabolites via phase II glucuronidation. Using cellular and *in vitro* CAR-coactivator interaction assays, deoxy-QHS has been shown to be an agonist of CAR1/3 and PXR, and it demonstrated induction of CYP2B6 and CYP3A4 expression in primary human hepatocytes [20]. The increased AUC ratio of two phase II metabolites (M4 and M5) to their respective phase I metabolites (M1 and M2) gave the direct evidence of auto-induction of phase II metabolism for QHS. These results also suggested that repeated doses would lead to a lower relative bioavailability of QHS and potential lower concentration level of its phase I metabolites. If a third daily dose of QHS-PQ were to be given, its relative bioavailability would also be low, especially in children. However, a three-day QHS-PQ regimen (3.2 and 16.0 mg/kg/day of QHS and PQ, respectively) has been found to be both well tolerated and more effective than a two-day regimen [4], which suggested that the lower bioavailability of the parent drug QHS and potential lower concentration level of its phase I metabolites might not be of clinical significance in the treatment of malaria infections.

Due to induction of phase I/II metabolism for QHS, more attention should be paid to the potential effect of QHS-PQ on the activity of major CYPs and UGTs enzymes. CYP2B6 and CYP3A4 have been reported to be involved in the metabolism of QHS [14,15]. In primary human hepatocytes, QHS was found to induce the activity of CYP3A4 and CYP2B6 in primary human hepatocytes, whereas QHS was also a weak reversible inhibitor of CYP2B6 (*K*_i_ 4.6 μM) [13]. Previous *in vitro* studies implicated that induction of CYP2B6 and CYP3A4 was the underlying mechanism of the time-dependent pharmacokinetics of QHS [11-13]. The induction of CYP3A4 activity by QHS has been evaluated by the ratio of plasma *C*_OH-MDZ_ to *C*_MDZ_ at 4 hours [16] or using different probe drugs (omeprazole or cortisol) [17]. The effect of QHS on the activity of CYP2B6 has been studied in humans using mephenytoin as a probe. In the present work, the effect of QHS-PQ on CYP2B6 and CYP3A4 activity in healthy Chinese after two consecutive oral doses of QHS-PQ was investigated. Considering that there is no report of PQ affecting any of the enzymes studied, the change of enzyme activity after QHS-PQ pretreatment was supposed to be the contribution of QHS in the present study.

In order to obtain metabolic information on different enzymes in one study, a cocktail approach, which involves the administration of several probe drugs simultaneously, is usually applied. By using metabolic ratios such as the AUC of a metabolite to that of the parent drug in plasma as metrics, the activity of multiple enzymes can be estimated concurrently. A number of studies have used the cocktail methodology to investigate the activity of major CYP450 enzymes (CYP1A2, CYP2C19, CYP2D6, CYP2E1, and CYP3A) [16]. It should be pointed out that both pharmacokinetic and pharmacodynamic interactions between cocktail probe drugs should be taken into consideration. In the present study, it was of interest to include probes for CYP2B6 and CYP3A4, as these enzymes have been shown to metabolize QHS *in vitro*. BUP and MDZ are well-recognized markers for evaluation of CYP2B6 and CYP3A activity, respectively, and have been widely used both *in vitro* and *in vivo*. A cocktail including BUP (100 μM) and MDZ (2 μM) has been validated *in vitro*[13,21,22]. To evaluate the activity of these two enzymes in humans, the pharmacokinetic profiles of MDZ and BUP have been evaluated separately [23-25] or dosed on separate days [26] due to lack of literature data to support this cocktail.

In the present study, MDZ and BUP were dosed together as a cocktail, which was validated by comparison of the present metabolic capability data (AUC_metabolite_/AUC_parent drug_) with reported results obtained from each probe alone or validated cocktail drugs. After an oral administration of two probe drugs, the dose-normalized AUC of BUP (0.35 ± 0.23 h kg/L) and its metabolite OH-BUP (3.98 ± 1.45 h·kg/L) corresponded with reported data obtained in healthy Chinese [24], Korean [25] and Caucasian volunteers [26]. The metabolic capability of CYP2B6 was 13.4 ± 5.2, which was in agreement with reported data (6.3-15.3). After an oral dose of this cocktail, the dose-normalized AUCs of MDZ (1.0 ± 0.3 h·kg/L) and its metabolite OH-MDZ (0.32 ± 0.09 h·kg/L) were similar to the documented data obtained in healthy Chinese [23] and Caucasian volunteers [25]. The AUC ratio of OH-MDZ to MDZ was 0.34 ± 0.09, which did not show a big difference from reported data (0.22-0.30). No serious drug-related adverse events occurred during and after the investigation. All volunteers successfully completed the study. These results suggested that bupropion (75 mg) and midazolam (15 mg) could be included in the present cocktail to evaluate the enzyme activity of CYP2B6 and CYP3A4, respectively.

The enzyme activity of CYP2B6 (bupropion hydroxylation) increased 2.1-fold after two days’ oral intake of QHS-PQ compared with day -14, which suggested that two days’ regimen of QHS-PQ could induce the activity of CYP2B6. In a previous population pharmacokinetic study, the activity of CYP2B6 (*S*-mephenytoin *N*-demethylation) increased 1.9-fold after multiple administrations of QHS to CYP2C19-poor metabolizers [12]. It was found that QHS could induce the activity of CYP2B6 (E_max_ 1.9-fold and EC_50_ 0.6 μM) in primary human hepatocytes [13]. However, weak inhibition by QHS was also observed in HLMs. Taking the induction and inhibition into consideration, quantitative prediction of P450-mediated DDIs indicated auto-induction of QHS clearance, as a result of a 1.6-fold increase in CYP2B6 activity. Previous studies provided molecular mechanism of induction by QHS, which acted as a ligand of human PXR and CAR. QHS could activate both nuclear receptors and result in the induction of the expression of CYP2B6, CYP3A4, and MDR1 in primary human hepatocytes and in the human intestinal cell line LS174T [27].

MDZ is probably the best available probe to test CYP3A4 activity, even though it is not selective for CYP3A4 vs. CYP3A5. In the present study, the CYP3A activity increased 3.2-fold after two consecutive days of drug treatment, which indicated that CYP3A4 could be induced by QHS. After five days’ treatment by QHS, the plasma *C*_OH-MDZ_/*C*_MDZ_ at 4 h (CYP3A activity) has been found to increase 2.66-fold in healthy subjects [16], and the increased CYP3A activity (1.6-fold) was observed even after one single dose. The increased activity of CYP3A4 (E_max_ 3.5-fold and EC_50_ 5.9 μM) by QHS pretreatment was also observed in primary human hepatocytes [13]. However, another study showed CYP3A4-enzymatic activity (omeprazole and cortisol as probes) was not induced by QHS in humans [17]. The choice of *in vivo* probe substrates may lead to variable results. In addition, the discrepancy of CYP3A4 activity may be caused by the net effect of induction and inhibition by QHS, which has been observed in human liver microsomes at very high concentration (IC50s of ~50 μM) [13]. Quantitative prediction of P450-mediated DDIs indicated auto-induction of QHS clearance, as a result of a net 1.9-fold increase in CYP3A4. Out of expectation, increased concentration level was observed for both MDZ and its metabolite OH-MDZ. The increased AUC ratio of OH-MDZ to MDZ indicated the induction of CYP3A activity by QHS, and the increased AUC/dose of MDZ (1.6 ± 0.5 fold) was probably caused by inhibition of intestinal CYP3A4 enzyme.

The elimination of QHS in human liver microsomes has been reported to be mediated by CYP2A6 with a probable secondary contribution [14], and minor induction of mRNA expression of CYP2A6 (E_max_ 11.7-fold and EC_50_ 4.0 μM) was observed in primary human hepatocytes [13]. Induction of CYP2A6 (7-hydroxycoumarin and its glucuronide excreted in human urine) was found after QHS pretreatment for five days in a previous study [18] but not observed in another study using 7-hydroxycoumarin as metrics [16]. QHS did not show induction of UDP glucuroninosyltransferases in primary human hepatocytes, but induction of glucuronidation (the AUC ratio of 7-hydroxycoumarin glucuronide to 7-hydroxycoumarin) existed for QHS in healthy subjects [19]. Based on these previous studies, CYP2A6 and UGTs were not studied in the present work due to their limited contribution to the initial auto-induction metabolism of QHS.

With a potential for induction and inhibition of drug metabolism by QHS and the recommendation of QHS-based combination treatment as first-line therapy of falciparum malaria by WHO, the risk for drug-drug interactions resulting in a potentiated or diminished activity of other drugs substantially increases. Moreover, it is important to realize that humans differ from patients with regard to the health status, and thus some caution should be applied when extrapolating metabolism data of QHS from healthy subjects to patients. Further assessment of induction of CYP (particularly CYP2B6, CYP3A and CYP2A6) and UGT enzymes involved in the metabolism of QHS, is required to clarify the entire picture of the pharmacokinetics of QHS in patients and other species of humans.

## Conclusions

The results gave the direct evidence for the presence of auto-induction phase I/II metabolism of QHS in healthy subjects during the two-day oral doses of QHS-PQ (Artequick), a recently marketed ACT combination therapy. The auto-induction metabolism also existed for phase I metabolites of QHS. The CYPs (CYP2B6 and CYP3A4) activity was induced after two consecutive oral doses of QHS-PQ. From the point of auto-induction metabolism and potential drug-drug interactions, a two-day oral regimen for QHS-PQ seems more reasonable than the alternative three-day treatment recommended for other ACT by WHO. However, other factors (the effect of malaria infection, the low proportion of QHS in QHS-PQ, potential under-dosing of PQ, and potential drug resistance caused by not enough QHS) deserve further evaluation of theoretically more efficacious three-day QHS-PQ regimen.

## Abbreviations

QHS: Artemisinin; PQ: Piperaquine; ACT: Artemisinin combination therapy; DHA: Dihydroartemisinin; ARS: Artesunate; ARM: Artemether; BUP: Bupropion; MDZ: Midazolam; OH-BUP: Hydroxybupropion; OH-MDZ: Hydroxymidazolam; HLM: Human liver microsome; IS: Internal standard.

## Competing interests

The authors declare that they have no competing interests.

## Authors’ contributions

MZ performed the experiments and analysed the data. FZ, XL and AY helped in performing the experiments. JX designed the experiments, analysed the data, and wrote the paper. All authors read and approved the final manuscript.
